# Osteoblast-Chondrocyte Interactions in Osteoarthritis

**DOI:** 10.1007/s11914-014-0192-5

**Published:** 2014-01-24

**Authors:** David M. Findlay, Gerald J Atkins

**Affiliations:** Centre for Orthopaedic and Trauma Research, The University of Adelaide, Royal Adelaide Hospital, Level 4 Bice Building, Adelaide, South Australia 5000 Australia

**Keywords:** Osteoarthritis, Articular, Cartilage, Subchondral, Bone, Osteoblasts, Chondrocytes, Gene expression, Communication, Pathogenesis, Transforming growth factor, Sclerostin

## Abstract

There is now general agreement that osteoarthritis (OA) involves all structures in the affected joint, culminating in the degradation of the articular cartilage. It is appropriate to focus particularly on the subchondral bone because characteristic changes occur in this tissue with disease progression, either in parallel, or contributing to, the loss of cartilage volume and quality. Changes in both the articular cartilage and the subchondral bone are mediated by the cells in these two compartments, chondrocytes and cells of the osteoblast lineage, respectively, whose primary roles are to maintain the integrity and function of these tissues. In addition, altered rates of bone remodeling across the disease process are due to increased or decreased osteoclastic bone resorption. In the altered mechanical and biochemical environment of a progressively diseased joint, the cells function differently and show a different profile of gene expression, suggesting direct effects of these external influences. There is also ex vivo and in vitro evidence of chemical crosstalk between the cells in cartilage and subchondral bone, suggesting an interdependence of events in the two compartments and therefore indirect effects of, for example, altered loading of the joint. It is ultimately these cellular changes that explain the altered morphology of the cartilage and subchondral bone. With respect to crosstalk between the cells in cartilage and bone, there is evidence that small molecules can transit between these tissues. For larger molecules, such as inflammatory mediators, this is an intriguing possibility but remains to be demonstrated. The cellular changes during the progression of OA almost certainly need to be considered in a temporal and spatial manner, since it is important when and where observations are made in either human disease or animal models of OA. Until recently, comparisons have been made with the assumption, for example, that the subchondral bone is behaviorally uniform, but this is not the case in OA, where regional differences of the bone are evident using magnetic resonance imaging (MRI). Nevertheless, an appreciation of the altered cell function during the progression of OA will identify new disease modifying targets. If, indeed, the cartilage and subchondral bone behave as an interconnected functional unit, normalization of cell behavior in one compartment may have benefits in both tissues.

## Introduction

**Fig. 1:**
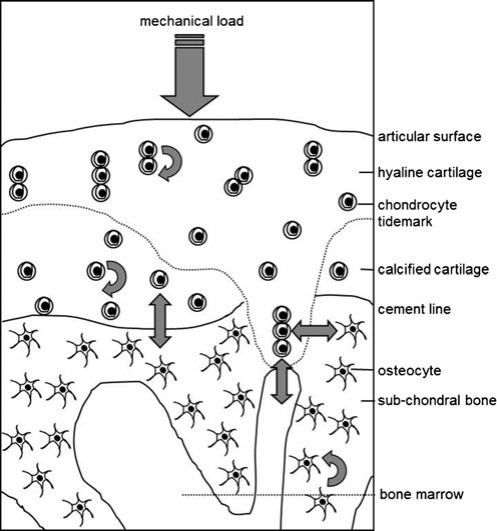
Schematic representation of an articular joint and the spatial relationship between chondrocytes in the hyaline and calcified cartilage and cells in the subchondral bone and bone marrow. Also depicted is the influence of repeated mechanical load and the response of chondrocytes with respect to production of matrix modifying factors such as MMPs (*curved arrows*), the response of load-sensing osteocytes with respect to the production of mediators such as prostaglandins and nitric oxide [12] (*curved arrows*), and the possibility of bidirectional communication between the cartilage and bone by the production of soluble mediators (*double headed arrow*). Not depicted are the possible additional influences of osteoblasts and osteoclasts involved in remodeling the subchondral spongiosa

There is now general agreement that osteoarthritis (OA) involves all structures in the affected joint, culminating in the degradation of the articular cartilage. This review focuses on cartilage and the underlying (subchondral) bone, since these compartments are intimately located (Fig. 1) and characteristic changes occur in parallel in these tissues across disease progression. Changes in both the articular cartilage and the subchondral bone are mediated by the cells in these two compartments: chondrocytes in the cartilage and osteoclasts, osteoblasts and osteocytes in the bone, whose primary roles are to maintain the integrity and function of these tissues. In response to the altered mechanical and biochemical environment of a progressively diseased joint, these cells function differently and show a different profile of gene expression, suggesting direct effects of these external influences. There is also ex vivo and in vitro evidence of chemical crosstalk between the cells in cartilage and subchondral bone, suggesting an interdependence of events in the two compartments. It is ultimately these cellular changes that explain the altered morphology of the cartilage and subchondral bone. An appreciation of the altered cell function during the progression of OA is likely to identify new targets for disease modification. If, indeed, the cartilage and subchondral bone behave as a functional unit, normalization of cell behavior in one compartment may have benefits in both tissues.

## Chondrocytes in OA

Articular cartilage and the disease processes that lead to its degeneration in OA have been intensively studied and extensively reviewed [1]. Articular chondrocytes are the only cell type resident in articular cartilage, and comprise a mere 2 %–5 % of its volume, which otherwise consists of a hydrated extracellular matrix of collagens (predominantly type II) and proteoglycans (importantly aggrecan). The role of the chondrocytes is to maintain the integrity of the cartilage by repairing damage due to minor or acute insults to the matrix. However, in the progression of OA, in which the biomechanical and biochemical environment of the cartilage is changed, characteristic changes also occur in the behavior of the chondrocytes. As recently reviewed [2], OA chondrocytes appear to resume the default differentiation pathway that is somehow blocked in articular cartilage, which leads to proliferation, hypertrophy, and apoptosis, as seen in the growth plate [3]. This more catabolic phenotype is characterized by decreased synthesis of the extracellular matrix and increased production of degradative enzymes, such as the matrix metalloproteinases (MMPs) and the aggrecanases. The repertoire of proteins produced by the cells changes toward that of hypertrophic chondrocytes, with prominent expression of type X collagen and MMP13, in turn driven by a reprogramming of the transcriptional machinery of the cell [2].

Although the initiating factors in OA have not been identified, mechanical damage is thought to interact with the effects of aging, in a genotype-dependent manner, to trigger local inflammation in the joint. Microarray analysis of adult human articular cartilage explants found that mechanical injury significantly regulated the expression of a large number of genes, collectively consistent with a reactivation of morphogenic pathways [4]. Foremost among the genes upregulated by injury was *WNT16*, which was barely detectable in preserved areas of cartilage in OA joints but was highly upregulated in areas of cartilage with moderate to severe cartilage damage. Interestingly, the wingless MMTV integration (Wnt) antagonist, sclerostin, was highly expressed only in focal areas of cartilage damage in sheep and murine models of OA, as well as in end-stage human OA cartilage [5]. In the same study, the gene encoding sclerostin, *SOST*, was also upregulated by interleukin (IL)-1 in articular chondrocytes. The addition of recombinant sclerostin protein to explant cultures inhibited the aggrecanase-inducing effects of IL-1, suggesting an anticatabolic role for sclerostin in OA cartilage [5].

Also suggesting an important role for the Wnt pathway in OA cartilage, over-expression of the Wnt antagonist, dikkopf (DKK)-1, protected against cartilage destruction in an animal model of OA [6•]. Further, Wnt-3A, which activates the Wnt pathway, induced the expression of MMP13 and a disintegrin and metalloproteinase with thrombospondin motifs (ADAMTS)-4 in primary chondrocyte cultures. IL-1 and tumor necrosis factor (TNF)-α have long been known to produce a catabolic phenotype in articular chondrocytes (reviewed in [7]) and may do so via the Wnt pathway. In an analogous tissue to articular cartilage, the intervertebral disc, TNF-α increased the expression of β-catenin and MMP13, and significantly inhibited the synthesis of type II collagen and proteoglycan, an effect that could be reversed by DKK1 [8]. While inflammatory cytokines have an essential role in the repair of all tissues, chronic exposure seems to compromise repair. In cartilage, persistent inflammation increases the remodeling rate of the extracellular matrix, leading to deficient matrix and poor quality cartilage. The links between biomechanical disruption, inflammation, and protease production by chondrocytes was demonstrated in a surgical model of mouse OA, in which genes such as IL-1, IL-6, MMP3, and ADAMTS 1, 4, and 5 were all upregulated after surgery. The expression of these genes was shown to be highly mechanosensitive because it was prevented by immobilization of the joint at the time of surgery [9].

Thus, although mechanical load is necessary for healthy cartilage, and limb immobilization leads to cartilage loss [10], load induced injury or chronic overloading of the joint can lead to changes in chondrocyte behavior that have long term consequences for matrix integrity. The question posed below is whether the chondrocyte reprogramming is at least partly due to signals from the subchondral bone, or vice versa, whether chondrocyte signals can influence cells in the bone.

## Osteoblasts in OA

Evidence from animal models of OA and from human bone samples obtained at surgery indicates altered bone remodeling in this disease. At the cellular level, remodeling is achieved by the actions of osteoclasts, which resorb bone, and osteoblasts, which are the bone forming cells. The activities of both of these cell types is regulated by osteocytes, embedded within the bone matrix [11, 12]. Overloading of the joints in OA seems to correspond with increased microdamage and microfracture in subchondral bone in the overloaded areas [13–15]. While it has been proposed that microdamage of bone protects articular cartilage due to enhanced energy absorption [14], it also promotes bone remodeling. Osteocytes detect damage of the mineralized bone matrix and direct its repair by initiating targeted osteoclastic resorption of the affected bone [16].

Recent evidence suggests that osteocytes produce the osteoclast differentiating cytokine, receptor activator of nuclear factor κB ligand (RANKL), in mature bone [17]. As recently discussed by Burr and Gallant [18••] the rate of bone remodeling changes across the course of the disease. Thus, increased remodeling, accompanied with increased vascularity, occurs in the subchondral bone in early OA, while late stage disease is characterized by reduced bone resorption with a bias toward bone formation. The changes in bone remodeling also vary spatially within the joint, for example medial versus lateral in the knee, but also more distally to the affected joint [19, 20]. Altered remodeling leads to altered bone structure, with increased BV/TV in cancellous bone and the formation of osteophytes [19, 21].

An increased trabecular number, decreased trabecular spacing, and reduced hardness of the bone in OA [22], due to decreased mineralization [23], characterize the subchondral bone, especially in zones underlying cartilage degeneration. Discrete zones of subchondral bone that can be imaged using magnetic resonance (MR), termed bone marrow lesions (BML), are frequently observed in both established OA and in early OA, but rarely in symptom free individuals [24, 25]. BMLs arise in regions of predicted high loading and contain abnormal bone, with areas of osteocyte death, areas of bone sclerosis with reduced mineral density [26]. Longitudinal studies have shown that BMLs occur adjacent to sites of current or future cartilage degeneration and are predictive of structural deterioration in knee OA [26–29] and future joint replacement [30].

The temporal and spatial changes in bone structure in OA are the result of altered cellular activity. The causes of this altered behavior are not yet known but are likely to be several. As stated, overloading of the joint can lead to an accumulation of damage in the bone matrix [31], which the cells attempt to resolve, and which is observed as increased remodeling in the bone. There is also some evidence for changes in perfusion of the bone, perhaps with episodes of ischemia [32], which could lead to osteocyte death followed by increased or decreased remodeling depending on whether blood supply is restored. What has been shown is that osteoblasts derived from OA bone display an altered phenotype and that gene expression in OA bone is different from that in either osteoporotic or normal bone.

For example, alkaline phosphatase and osteocalcin levels were found to be elevated in OA osteoblasts compared with normal osteoblasts, whereas osteopontin levels were similar [33]. In addition, osteoblasts from OA bone showed disturbed mineralization compared with control cells, with dramatically variable calcium: phosphate ratios compared with the value of approximately 1.6 for control osteoblasts and normal bone [34].

In addition, Couchourel et al [33] reported reduced mineralization by OA osteoblasts, accompanied by an elevated *COL1A1*:*COL1A2* mRNA ratio, similar to the differential expression of these genes in OA bone [35]. Interestingly, OA osteoblasts produce more TGFβ1 than normal osteoblasts, and inhibiting TGFβ1 in OA osteoblasts corrected the abnormal *COL1A1*:*COL1A2* ratio and increased cell mineralization [35]. The increased production of transforming growth factor (TGF) β1 by OA cells induced increased levels of DKK-2 and silencing of either TGFβ or DKK2 in these cells was found to normalize the OA mineralization phenotype [36]. Massicotte et al [37] reported two subgroups of osteoblasts derived from OA subchondral bone, based on production of IL-6 and prostaglandin E_2_, while TGFβ1 levels were increased in all osteoarthritic osteoblasts compared with normal. Kumarasinghe et al [38] performed analysis of gene expression in primary osteoblasts derived from OA and control femoral bone across differentiation. These studies showed that the dysregulated expression of *TWIST1*, *TGFβ1*, and *SMAD3* mRNA observed previously in OA bone is also present in OA osteoblasts when these cells are cultured ex vivo, and proposed that at least part of the etiology of OA is due to altered intrinsic properties of the osteoblasts [34]. A recent report confirmed the high concentrations of TGFβ1 in subchondral bone in human and mouse OA, and went on to show that transgenic overexpression of *TGFβ1* in the subchondral bone actually induced OA [39••]. Significantly, inhibition of TGFβ specifically in the subchondral bone improved the bone architecture in the anterior cruciate ligament transection mouse model of OA and attenuated the degeneration of articular cartilage and the percentage of chondrocytes in the cartilage expressing MMP13 and type X collagen. These positive effects of TGFβ1 inhibition were not seen with systemic approaches to inhibit TGFβ activity, since TGFβ is required for cartilage homeostasis and the systemic, but not targeted, TGFβ inhibition blocked TGFβ signaling in articular cartilage. The authors therefore concluded that high concentrations of TGFβ in the subchondral bone induced abnormal bone formation and the development of OA [39••]. The above comments are generalities because no data are available regarding the behavior of osteoblast-lineage across the course of OA or, in any systematic way, from different zones in a diseased joint.

## Crosstalk Between Chondrocytes and Osteoblasts in OA

It is possible that chondrocytes and cells of the bone are responding independently to the same environmental cues, which is exhibited in an altered cell phenotype in OA. Alternatively, cellular changes in one or both compartments may influence cells in the other compartment. There is mounting evidence, both ex vivo and in vivo, that chondrocytes and osteoblasts are able to influence each other. For example, in the well-established rat model of OA, in which monosodium iodoacetate (MIA) is injected intra-articularly, it is the cartilage that is initially exposed to the drug. However, the model shows significant bone loss after only 2 weeks, followed later by increased trabecular thickness and the presence of subchondral bone sclerosis, cysts, and osteophytes, in parallel with cartilage degradation [40•]. Conversely, over-expression of the EPHB4 receptor specifically in osteoblasts and, therefore, subchondral bone, exerted a protective effect against OA in mice, induced by medial meniscus destabilization [41•]. Since the subchondral bone was also preserved in the transgenic animals, there are potentially mechanical as well as biochemical explanations for this finding, which nonetheless demonstrates the interdependence of the two tissue compartments.

There is good evidence that cartilage loss occurs in the same regions of the joint as the changes in the subchondral bone [42]. It is, therefore, perhaps not surprising that in animal models of OA, applying treatments that are thought to specifically target the subchondral bone can ameliorate or prevent disease progression. For example, in rat models of knee OA, the bisphosphonate, alendronate, suppressed both subchondral bone resorption and the development of OA in the knee joint [43]. Similarly, calcitonin reduced the levels of circulating bone turnover markers and the severity of OA lesions dog models of OA [44, 45]. Systemic injection of osteoprotegerin (OPG), to block RANKL-mediated bone remodeling in a mouse menisectomy model of OA, increased bone volume in the operated and nonoperated knee bones, as would be expected, but also dramatically protected from the meniscectomy-related OA [46]. OPG also significantly reduced ADAMTS-4 and ADAMTS-5 expression in the articular chondrocytes following meniscectomy, although not to control levels. In a similar mouse model, pamidronate dramatically preserved the bone mass and reduced the OARSI score, at the same time almost normalizing the expression of ADAMTS-4 and ADAMTS-5 in the overlying joint cartilage [47]. In a human study, strontium ranelate, which is thought to act on bone to reduce turnover, significantly reduced CTX-II, a marker of cartilage degradation, in subjects with a history of OA [48].

There is accumulating ex vivo and in vitro evidence that events in the subchondral bone have a direct effect on the overlying cartilage. Amin et al [49] reported on chondrocyte survival in bovine cartilage explants in culture, which included or excluded the underlying subchondral bone. It was found that excision of subchondral bone from articular cartilage resulted in an increase in chondrocyte death at 7 days, mainly in the superficial zone of the cartilage. However, the presence of the excised subchondral bone in the culture medium abrogated this increase in chondrocyte death, most likely due to soluble mediator(s) released from the subchondral bone. Sanchez et al [50] described a coculture system, in which osteoblasts derived, respectively, from ‘sclerotic’ or ‘nonsclerotic’ regions of human subchondral bone in OA were separated by a membrane from chondrocytes in alginate beads. Clear evidence was presented for cross-talk between the cells, with chondrocytes cultured in the presence of ‘sclerotic’ osteoblasts, but not ‘nonsclerotic’ osteoblasts, exhibiting reduced production of aggrecan and increased expression of MMP3 and MMP13. This influence was magnified when the osteoblasts were pretreated with inflammatory mediators IL-1, IL-6, or oncostatin M (OSM). These data suggest the possibility at least of disease exacerbating changes in chondrocytes proximal to phenotypically OA osteoblasts. The authors further speculate that the IL-1, IL-6, or OSM, which are overproduced by OA chondrocytes, could create a feedback loop by acting on nearby osteoblasts. Although osteocytes are the most abundant cell type in bone and are the primary mechanosensing cell type [11, 12], and interactions between chondrocytes and osteocytes are therefore likely of primary importance, very little has been reported concerning this interaction. In one such study, however, Priam et al [51••] conducted experiments in which mouse calvarial osteoblast/osteocytes were subjected to cyclic compression and mouse articular chondrocytes were exposed to the osteoblast conditioned medium. Conditioned medium from the compressed cells caused a dramatic up-regulation of MMP3 and MMP13 expression in the chondrocytes and down-regulated expression of aggrecan and type II collagen. The study identified 14-3-3ε as a soluble mediator for communication between the osteoblasts/osteocytes and chondrocytes [51••].

The above experiments demonstrate the potential for molecular crosstalk between osteoblasts/osteocytes and chondrocytes in vivo but it has long been thought that there was little possibility of communication between subchondral bone and articular cartilage. However, Imhof et al [52] described the dense subchondral vasculature in close proximity to the cartilage and the micro-channels that penetrate the subchondral mineralization zone and permit communication between the bone and the cartilage. Imhof et al [52] have further claimed that more than 50 % of the glucose, oxygen, and water requirements of cartilage are provided by perfusion from the subchondral vessels. In support of this, experimentally induced hypoxia of the femoral head led to cell death in the bony epiphysis and in the deep layer of the overlying cartilage [53]. Indeed, perfusion abnormalities have been identified in OA, in particular in zones of the subchondral bone identified by MR imaging as bone marrow ‘edema’ or bone marrow lesions [54].

As discussed above, these zones underlie regions of degraded cartilage or predict cartilage degradation [27–29]. In elegant experiments using fluorescent dyes, Pan et al [55] showed the diffusion of these small molecules between the bone marrow and the articular space. These observations suggest the possibility of direct signaling between subchondral bone and articular cartilage, at least for small molecules. The authors further suggested that the two compartments form a functional unit both mechanically and biochemically, which may play a role in the maintenance and degeneration of the joint. These results were consistent with several other observations. The ultrastructure of the interface between the subchondral bone and calcified cartilage provides numerous vascular (Haversian) canals, by which these tissues could communicate [56].

A more recent study of the human chondro-osseous junction revealed a hitherto unappreciated complexity [57]. For example, uncalcified cartilage was sometimes seen dipping through the calcified cartilage into bone and marrow spaces. The authors commented that this proximity of hyaline cartilage and marrow spaces could provide a molecular diffusion pathway, which may have nutritional, metabolic, and biomechanical roles. In addition, since this interface is profoundly affected by OA, these areas could enable trafficking of humoral mediators between these tissues (Fig. 1). Finally, a large increase in subchondral plate porosity was recently shown during disease development in a mouse model of OA [58], and it was again suggested that this may enhance mutual interaction between the bone and cartilage compartments. It remains to be formally demonstrated that cytokine-sized molecules can traverse between bone and cartilage in either healthy or diseased joints. However, larger molecules than previously thought are able to traverse osteocyte canaliculi, and this transport is increased by bone loading [59]. Similar principles may apply to transport through the porosity linking these compartments, which potentially includes the vasculature, the canalicular porosity and the pores in the subchondral bone plate and the calcified cartilage. With respect to the cartilage, diffusion of cell mediators has been well demonstrated. For example, insulin-like growth factor (IGF) has important roles in cartilage but is produced in low amounts only and is largely transported into cartilage from the circulation [60•]. This transport occurs partly by diffusion across a concentration gradient but, in addition, cyclic loading has been shown to enhance the transport of this relatively large molecule by advection [61].

## Conclusions

OA is caused by factors, some known and some unknown, which result in an altered phenotype of osteoblasts and osteocytes in bone and chondrocytes in cartilage. There is much interest as to whether there is crosstalk between cells in these compartments, since there is now good evidence that these cells can signal in vitro in ways that can promote chondrocyte catabolism. There is also good evidence that small molecules can traverse between these tissue compartments in situ but this evidence is lacking for larger cell mediators. The importance of this issue is that directing treatment to either compartment may provide a circuit breaker in OA to prevent or slow the progression of this condition.
